# Adhesion of *Candida Albicans* to digital versus conventional acrylic resins: a systematic review and meta-analysis

**DOI:** 10.1186/s12903-024-04083-2

**Published:** 2024-03-04

**Authors:** Mohammed Nasser Alhajj, Esam Halboub, Norlela Yacob, Sadeq Ali Al-Maweri, Siti Fauzza Ahmad, Asja Celebić, Hesham M. Al-Mekhlafi, Nosizana Mohd Salleh

**Affiliations:** 1 Department of Restorative Dentistry, Faculty of Dentistry, Universiti Malaya, 50603 Kuala Lumpur, Federal Territory of Kuala Lumpur Malaysia; 2 Department of Maxillofacial Surgery and Diagnostic Sciences, College of Dentistry, Jazan University, Jazan, Saudi Arabia; 3 Department of Oral Medicine, Oral Pathology and Oral Radiology, Faculty of Dentistry, Sana’a University, Sana’a, Yemen; 4 Department of Conservative Dentistry & Prosthodontics, Faculty of Dentistry, Universiti Sains Islam Malaysia, Negeri Sembilan, Malaysia; 5 College of Dental Medicine, QU Health, Qatar University, Doha, Qatar; 6 Department of Removable Prosthodontics, Faculty of Dentistry, University of Zagreb, Zagreb, Croatia; 7 Department of Parasitology, Faculty of Medicine, Universiti Malaya, Kuala Lumpur, Federal Territory of Kuala Lumpur Malaysia

**Keywords:** Candida Albicans, Digital denture, CAD-CAM, Acrylic resin, Prosthodontics

## Abstract

**Background:**

The present systematic review and meta-analysis investigated the available evidence about the adherence of *Candida Albicans* to the digitally-fabricated acrylic resins (both milled and 3D-printed) compared to the conventional heat-polymerized acrylic resins*.*

**Methods:**

This study followed the guidelines of the Preferred Reporting Items for Systematic Review and Meta-analyses (PRISMA). A comprehensive search of online databases/search tools (Web of Science, Scopus, PubMed, Ovid, and Google Scholar) was conducted for all relevant studies published up until May 29, 2023. Only in-vitro studies comparing the adherence of *Candida albicans* to the digital and conventional acrylic resins were included. The quantitative analyses were performed using RevMan v5.3 software.

**Results:**

Fourteen studies were included, 11 of which were meta-analyzed based on Colony Forming Unit (CFU) and Optical Density (OD) outcome measures. The pooled data revealed significantly lower candida colonization on the milled digitally-fabricated compared to the heat-polymerized conventionally-fabricated acrylic resin materials (MD = − 0.36; 95%CI = − 0.69, − 0.03; *P* = 0.03 and MD = − 0.04; 95%CI = − 0.06, − 0.01; *P* = 0.0008; as measured by CFU and OD respectively). However, no differences were found in the adhesion of *Candida albicans* between the 3D-printed digitally-fabricated compared to the heat-polymerized conventionally-fabricated acrylic resin materials (CFU: *P* = 0.11, and OD: *P* = 0.20).

**Conclusion:**

The available evidence suggests that candida is less likely to adhere to the milled digitally-fabricated acrylic resins compared to the conventional ones.

**Supplementary Information:**

The online version contains supplementary material available at 10.1186/s12903-024-04083-2.

## Background

The rapid and huge progress in all aspects of technology imposes a growing interest in using computer-aided design and computer-aided manufacturing (CAD/CAM) for fabricating removable digital dentures [1]. According to the Glossary of Digital Dental Terms, “digital denture” refers to a dental prosthesis fabricated through automation using CAD/CAM and computer-aided engineering [2]. Digital dentures can be fabricated using subtractive manufacturing, whereby the bulk denture resin is removed according to the designated denture in a milling machine [3]. In contrast, additive manufacturing involves incrementing the resin as layers with photopolymerization depending on the type of 3D printer, either by stereolithography or digital light processing (DLP) [4, 5]. Despite the differences in fabrication techniques, the chemical composition of the resins used is almost similar [6].

Studies have reported that the application of milling and 3D printing in the fabrication of dentures results in excellent surface adaptation, comparable strength to the conventional ones, and good clinical outcomes [7], with high levels of patient satisfaction [8]. According to the dental literature, the milled resin exhibits more stability and less polymerization shrinkage compared to the 3D-printed resin [9]. The milled acrylic resin is made of pre-polymerized blocks which minimize or even lack the after-processing shrinkage, while in the 3D-printed resin, the polymerization process may be less controllable, which can lead to variations in shrinkage. In addition, the surface properties of the milled dentures are superior to those of the 3D-printed dentures [10]. However, several factors such as the type of a printer, build angulation, and a layer thickness may affect the surface and mechanical properties [11].

Denture-related stomatitis (DS) is a primary concern when it comes to removable prostheses. The prevalence of DS among denture wearers ranges from 15 to 70% [12]. It is more common among the elderly population, and can, interestingly, increase the risk of systemic infection [13]. DS is a multifactorial disease with the predominantly associated factors are, among others, poor denture hygiene, continuous night-time denture wearing, and Candida infection [12]. DS can develop faster than previously reported, even with new dentures; continued denture wearing and poor cleaning of dentures revealed a considerable impact on DS onset, with *Candida albicans* (*C. albicans)* as the most identified kind of yeast [14]. Indeed, Candida adhesion and proliferation on the surface of the acrylic denture base can lead to inflammation of the oral tissue, especially of the denture-fitting surface [15, 16]. This issue is controversial; however, recent studies suggested that the adherence of multiple species of microbes on the denture surface leads to the pathogenesis of denture stomatitis [17–19].

*Candida albicans* is the key pathogen implicated in the development of denture stomatitis [20, 21]. In this context, several studies have reported lower Candida adhesion to milled dentures compared to the conventional ones [18, 22, 23], with inconsistent results regarding the 3D-printed dentures [19, 24–26]. Other studies suggested that *C. albicans* has almost similar adherence on the 3D-printed and the milled denture surfaces [11, 18]. However, it is worth to note that the milled dentures have less adherence affinity, thus reducing the risk of DS occurrence [27], while the adherence affinity is high on the 3D-printed dentures [28], especially if the print orientation is not optimized, thus increasing the risk of DS [11]. Collectively, although the digital dentures have shown promising results, the long-term outcomes and clinical performance are still lacking. Moreover, since the digital dentures are new to the field, there has been no concrete evidence on the extent of Candida adherence on their surfaces so far [22, 23, 27–36]. Therefore, this systematic review and meta-analysis aimed to assess the available evidence regarding Candida adherence to the digitally-fabricated acrylic resins (both milled and 3D-printed) in comparison to conventional heat-polymerized acrylic resins.

## Methodology

### The registration and focused question

This systematic review followed the Preferred Reporting Items for Systematic Reviews and Meta-Analyses (PRISMA) guidelines [37]. The protocol of this systematic review was registered in the PROSPERO registry (ID: CRD42023390907). The focused research question is, “Does digital acrylic resin (milled and 3D-printed) have higher fungal adherence affinity than conventional heat-polymerized acrylic resins?”

### Eligibility criteria

The inclusion criteria were as follows: a) Controlled in-vitro studies that compared candidal colonization on the digital (milled or 3D-printed) acrylic with the conventional heat-polymerized acrylic resins, and b) Articles published in English with no time limits. The exclusion criteria were: review articles, editorials, commentaries, abstracts, case reports, uncontrolled studies, in-vivo studies, and studies published in a language other than English. The PICOS framework was used to formulate the research question as follows: Population (P): digital acrylic resin (milled or 3D-printed); Intervention (I): exposure to candidal culture; Comparator (C): conventional heat-polymerized acrylic resin; Outcome (O): candida growth; and study design (S): a controlled in-vitro study.

### Search strategy and information sources

Two investigators conducted an independent, yet meticulous and thorough search of multiple online databases/search engines (Web of Science, Scopus, PubMed, Ovid, and Google Scholar) for all relevant studies published up until May 29, 2023. Different combinations of the following keywords were used with the aid of the Boolean operators (AND, OR): (“CAD-CAM denture” OR “CAD/CAM denture” OR “digital denture” OR “3d printed denture” OR “printed denture” OR “printed resin” OR “milled denture” OR “milled resin” OR “conventional heat-polymerized acrylic resin” OR “conventional resins” OR “conventional denture” OR “heat-polymerized acrylic resin” OR “heat-polymerized acrylic denture”) AND (“antimicrobial” OR “adhesion” OR “antifungal” OR “candida” OR “colonization”). Table 1 in Supplementary file 1 provides a detailed description of the search strategy in the different databases/search engines.

### Screening and selection process

The studies retrieved were exported to the EndNote program, and the duplicates, if any, were removed. Two investigators independently screened the remaining studies, based on the title and abstract, to identify the relevant articles. The full-texts of the potentially relevant studies were retrieved and assessed for inclusion. In addition, a manual search of the reference lists of the included studies was performed to identify any additional relevant studies.

### Data extraction

Two reviewers extracted the following data independently: the name of the author(s), publication year, type of acrylic resins used, number of samples per group, dimensions of the specimen, type, and number of microbial isolates, time, temperature, and concentration of candidal exposure, and the measurement method of efficiency. Concerning Wei et al. study [36], the numerical data were extracted from their figures using a semi-automated online tool called “WebPlotDigitizer”, available at https://apps.automeris.io/wpd/ (Supplementary Fig. 1). Concerning Koujan et al. study [27], the standard errors of the means were converted to standard deviations following the formula: $SE= SD/\sqrt{n}$. With regard to Linder study [34], the differences between the groups were not reported completely; so that the mean, SD, and N were used for pairwise comparisons between the digital and conventional groups using GraphPad Prism 9.5.0 (GraphPad Software, San Diego, CA, USA) to obtain clear comparison results (Supplementary file 2). The extracted data were then tabulated for further analysis.

### Risk of bias assessment

Two reviewers independently and thoroughly assessed the risk-of-bias of the included studies utilizing the QUIN tool. Discrepancies, if any, were resolved by group discussion. The QUIN tool consists of 12 criteria recommended for assessing the risk-of-bias of in-vitro studies conducted in dentistry [38]. According to the nature of the included studies, two criteria, namely “Detailed explanation of sampling technique” and “Randomization”, were excluded as being inapplicable. Each criterion is given a score of 2 points if adequately specified, 1 point if inadequately specified, and 0 points if not specified. The inapplicable criteria were excluded from the calculation. The criteria individual scores were then added to obtain a total score for a given in-vitro study. This total score was recalculated out of 100% according to the following formula:

$$\textrm{Final}\ \textrm{score}=\frac{\textrm{Total}\ \textrm{score}\times 100}{2\times \textrm{number}\ \textrm{of}\ \textrm{criteria}\ \textrm{applicable}}$$

The included studies were qualified as having “low risk of bias”, “medium risk of bias”, or “high risk of bias” if they scored > 70%, from 50 to 70%, or < 50%, respectively.

## Statistical analysis

The quantitative analyses were conducted using Review Manager (RevMan) v5.3 software program for Windows. Only studies that used CFU or OD values as outcome measures were included in the analysis. The pooled mean differences (MD) were calculated as a summary estimate between the digital and the conventional resins. According to the type of outcome measure, two separate meta-analyses were conducted; one for the CFU outcomes, and the other for OD outcomes. Additionally, subgroup analysis was utilized for the 3D-printed and milled resins groups separately. The summary estimates were reported along with their corresponding 95% confidence intervals (CIs). Heterogeneity among the studies was evaluated using the *χ*^2^ test I^2^ statistic. A fixed-effect model was applied for insignificant heterogeneity (I^2^ ≤ 50%), while a random-effect model was used for significant heterogeneity (I^2^ > 50%). In addition, StataMP-64 was used for Egger’s test to quantitatively identify the publication bias of the included studies. The significance level was set at a *P*-value < 0.05.

## Results

### Study selection

**Fig. 1:**
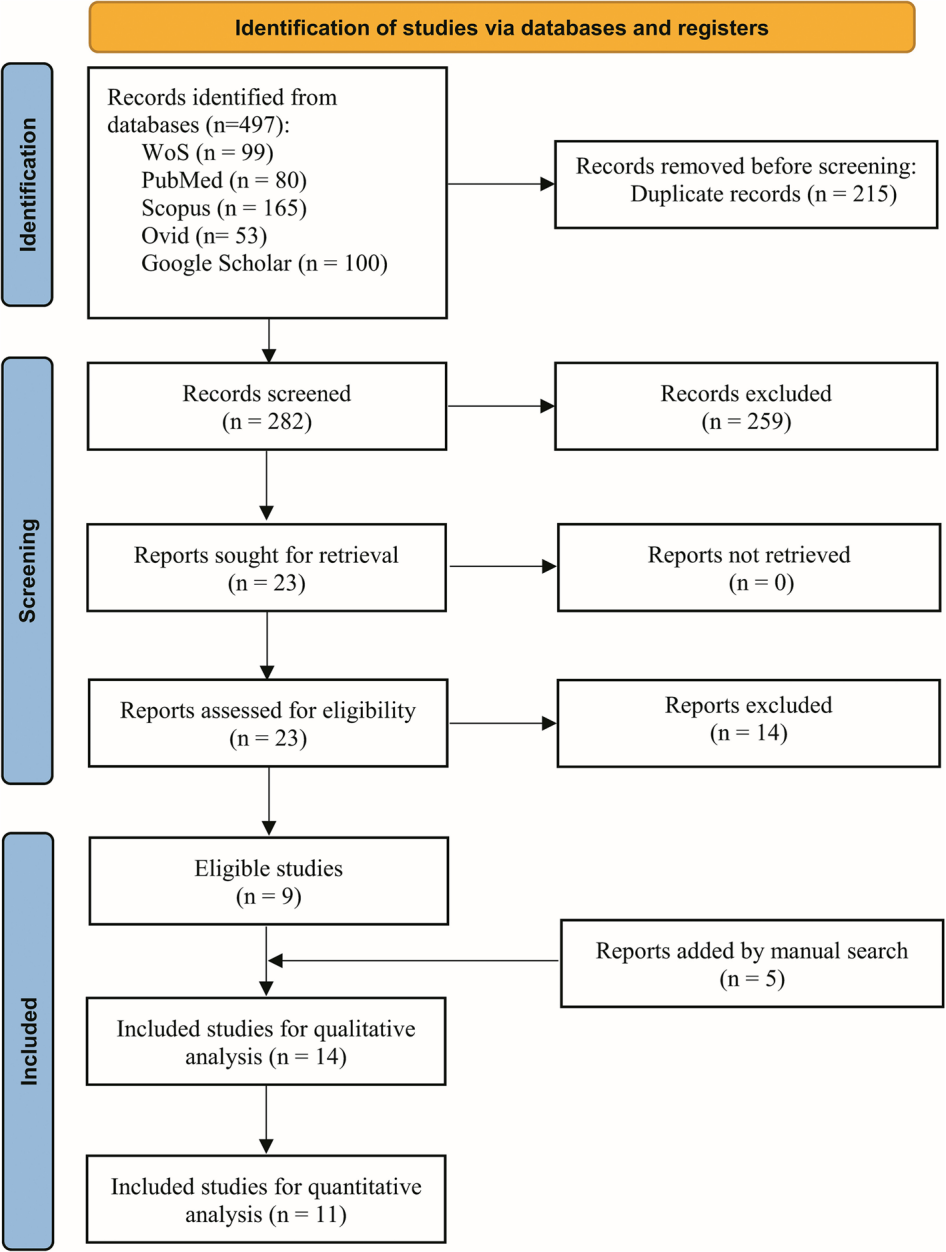
Flow chart of the search strategy

Figure 1 depicts the search strategy employed following the PRISMA guidelines. The online search yielded a total of 497 studies; 215 of which were removed owing to being duplicates. Among the remaining 282 studies, 259 were excluded based on screening their titles and abstracts. The full-texts remaining and potentially relevant 23 studies were retrieved and assessed for eligibility. Of those, 14 studies were excluded for various reasons detailed in the Supplementary file 1: Table 2. A manual search yielded an additional 5 articles meeting the inclusion criteria. As a result, 14 studies were included for qualitative analysis, and 11 of which were included in the quantitative analysis.

### Characteristics of the included studies

Table 1 presents the general characteristics of the included studies. There were 14 studies, including 35 independent comparison groups, published between 2014 and 2023. Five studies [22, 23, 33, 35, 36] used only milled acrylic resin, two studies [32, 40] used only 3D-printed acrylic resin, while 7 studies [27–31, 34, 39] used both milled and 3D-printed acrylic resins. The sample sizes varied from 4 to 15 bars/discs with different dimensions. Regarding the microbial isolate, 2 studies [22, 33] used four strains of *C. albicans*, while the other 12 studies [23, 27–32, 34–36, 39, 40] used only one strain. The measurement methods for the outcomes also varied across the included studies: 7 studies [22, 29–32, 34, 40] used CFU/ml (or log CFU/ml), 3 studies [27, 33, 39] used OD, and one study [36] used both CFU/ml and OD. Meanwhile, one study each used adhesion percent [35], cell count per field [23], and microbial cell count [28]. The numerical data which extracted from the included studies and utilized for the meta-analyses are shown in Table 2.

**Table 1:** Characteristics of the included studies

Author, Year (Country)	Digital acrylic resin	Conventional acrylic resin	Sample size (dimensions in mm)	Microbial isolate (number of strains)	Measurement unit	Time of exposure Temperature (Concentration)	Polish/non-polish Saliva coated Stationary/dynamic	Main outcome
Makke, 2014 (USA) [35]	Milled (AvaDent)	Eclipse Light-cured Lucitone FRS flexible SR Ivocap High Impact Nature-CRYL Pour DC Acrylic S.P. Clear Ivocap	15 bars (5 × 15 × 2 mm)	*Candida albicans* (1)	Adhesion percent	1 hrs at 30 °C (1 × 10^6^ cells/mL)	Non-polish Saliva coated Stationary	A significant lower candida colonization in CAD-CAM group (*P* < 0.05).
Al-Fouzan et al., 2017 (KSA) [22]	Milled (VivaDent)	Heat-polymerized AR	10 discs (10 × 3 mm)	*Candida albicans* (4)	CFU/mL	90 min at 37 °C (1 × 10^7^ cells/mL)	Polished NR Dynamic	A significant lower Candida in the milled group (*P* < 0.05).
Murat et al., 2019 (Turkey) [23]	Milled (M-P-M) Milled (Polident) Milled (AvaDent)	Heat-polymerized AR	10 discs (10 × 2 mm)	*Candida albicans* (1)	cell count per field	2 hrs at 37 °C (NA)	Finish at 800-grit-saliva coated/non-coated Dynamic	A significant lower Candida in the milled group (*P* < 0.05).
Jung, 2020 (USA) [31]	Milled (AvaDent) 3D printed (Dentca) DLP printed (Lucitone)	Heat-polymerized AR	12 bars (10 × 10 × 2 mm)	*Candida albicans* (1)	CFU/mL	24 hrs at 37 °C (1 × 10^7^ cells/mL)	Non-polish Non-coated Stationary	No significant differences between the two groups.
Meirowitz et al., 2021 (Israel) [28]	Milled (Vira Vionic) 3D-printed (Detax)	Heat-polymerized AR Chemically-polymerized AR	6 discs (12 × 2 mm)	*Candida albicans* (1)	Microbial cell count	4 hrs at 37 °C (1 × 10^6^ cells/mL)	Polished Mucin coated Stationary	Significantly lower colonization in milled resin than conventional. 3D showed the highest colonization.
Freitas et al., 2023 (Brazil) [30]	Milled (AvaDent) 3D-printed (Yller)	Heat-polymerized AR Microwave-polymerized AR	9 discs (10 × 3 mm)	*Candida albicans* (1)	log CFU/mL	48 hrs at 37 °C (1 × 10^7^ cells/mL)	Polished Non-coated Stationary	Milled digital showed lower colonization than conventional denture (*P* < 0.05). Yet, 3D dentures showed higher colonization than conventional.
Koujan et al., 2022 (USA) [27]	Milled (AvaDent) 3D-printed (Dentca)	Heat-polymerized AR	10 bars (10 × 10 × 2 mm)	*Candida albicans* (1)	OD value	16 hrs at 37 °C (NA)	Polish Non-coated Shaker-dynamic	No significant differences between the milled and conventional, but significantly higher colonization in 3D digital.
Larijani et al., 2022 (Iran) [33]	Milled (Glazed) Milled (High polished)	Heat-polymerized AR Chemically-polymerized AR	14 discs (10 × 1 mm)	*Candida albicans* (4)	OD value	48 hrs at 37 °C (1 × 10^8^ cells/mL)	Polished Non-coated Stationary	No significant differences between the glazed milled and conventional group. However, significantly lower in polished milled compared to the conventional.
Linder, 2022 (USA) [34]	Milled (Ivotion) 3D-printed (Dentca) DLP printed (Lucitone) DLP printed (Envision)	Heat-polymerized AR (Injected) Heat-polymerized AR (Compressed)	6 discs (10 × 2 mm)	*Candida albicans* (1)	log CFU/mL	48 hrs at 37 °C (NA)	Polished Non-coated Stationary	No significant differences between 3D group and conventional group. Yet, significantly lower DLP group than the conventional.
Wei et al., 2022 (China) [36]	Milled (Organic)	Heat-polymerized AR (Compressed)	4 discs (10 × 2 mm)	*Candida albicans* (1) *Staphylococcus aureus* (1) *Streptococcus mutans* (1)	log CFU/ml OD value	24 hrs at 37 °C (1 × 10^7^ cells/mL)	Polished Saliva coated Stationary	A significant lower candida colonization in the milled group (*P* < 0.05).
Alfouzan et al., 2023 (KSA) [29]	Milled (IvoBase) 3D-printed (NextDent)	Heat-polymerized AR	10 discs (10 × 3 mm)	*Candida albicans* (1) *Streptococcus mutans* (1)	CFU/mL	72 hrs at 37 °C (1 × 10^−3^ cells/mL)	Polished Non-coated Shaker -dynamic	No significant differences between the two groups.
Khattar et al., 2023 (KSA) [32]	3D-printed (NextDent)	Heat-polymerized AR	10 discs (15 × 2 mm)	*Candida albicans* (1)	CFU/mL	24 hrs at 37 °C (NR)	Polished Non-coated Stationary	No significant differences between the two groups.
Osman et al., 2023 [39]	3D-printed (NextDent) Milled (Opera)	Heat-polymerized AR	9 specimens Not specific shape	*Candida albicans* (1)	OD value	24 hr. at 37’C (1 × 10^6^ cells/mL)	Non-polished Non-coated Dynamic-shaker 150 rpm	Milled digital showed lower colonization than conventional denture (*P* < 0.05). Yet, 3D-printed resin showed higher colonization than conventional.
Teixeira et al., 2023 [40]	3D-printed (Cosmos)	Heat-polymerized AR	9 discs (9 × 1 mm)	*Candida albicans* (1) Candida Glabrata (1) *Streptococcus mutans* (1)	CFU/mL	90 min at 37’C (1 × 10^6^ cells/mL)	Polished Non-coated Dynamic-shaker 750 rpm	3D-printed resin showed higher colonization than conventional.

**Table 2:** The extracted means and SDs of the fungal colonization in different output units (CFU/ml or OD value), and the main conclusions of the studies subjected to quantitative analysis

Study	Digital acrylic resin		Conventional acrylic resin		Conclusion
	Type	Mean ± SD	Type	Mean ± SD	
Al-Fouzan et al., 2017 [22]	Milled (VivaDent)	1.1 × 10^3^ ± 6.0 × 10^2^	HPAR	2.3 × 10^3^ ± 8.4 × 10^2^	Dig. < Conv.
	Milled (VivaDent)	2.1 × 10^3^ ± 8.7 × 10^2^	HPAR	5.4 × 10^3^ ± 1.6 × 10^2^	Dig. < Conv.
	Milled (VivaDent)	1.2 × 10^3^ ± 8.8 × 10^2^	HPAR	2.0 × 10^3^ ± 9.7 × 10^2^	Dig. < Conv.
	Milled (VivaDent)	1.5 × 10^3^ ± 7.2 × 10^2^	HPAR	2.4 × 10^3^ ± 1.1 × 10^3^	Dig. = Conv.
Jung 2020 [31]	Milled (AvaDent)	100.92 ± 62.80	HPAR	109.75 ± 52.32	Dig. = Conv.
	3D-printed (Dentca)	81.70 ± 47.17			
	3D-printed (Lucitone)	65.58 ± 34.81			
Freitas et al., 2023 [30]	Milled (AvaDent)	3.74 ± 0.57	HPAR	5.12 ± 1.01	Dig. < Conv.
	3D-printed (Yller)	5.77 ± 0.36			Dig. > Conv.
Linder 2022 [34]	Milled (Ivotion)	4.85 ± 0.19	HPAR (Injected)	4.90 ± 0.05	Dig. = Conv.
	3D-printed (Dentca)	4.91 ± 0.13			Dig. = Conv.
	3D-printed (Lucitone)	4.28 ± 0.13			Dig. < Conv.
	3D-printed (Envision)	4.25 ± 0.39			Dig. < Conv.
	Milled (Ivotion)	4.85 ± 0.19	HPAR (Compressed)	4.84 ± 0.04	Dig. = Conv.
	3D-printed (Dentca)	4.91 ± 0.13			Dig. = Conv.
	3D-printed (Lucitone)	4.28 ± 0.13			Dig. < Conv.
	3D-printed (Envision)	4.25 ± 0.39			Dig. < Conv.
Wei et al., 2022 [36]	Milled (Organic)	6.36 ± 0.22	HPAR	7.06 ± 0.13	Dig. < Conv.
	Milled (Organic)	0.04 ± 0.04	HPAR	0.21 ± 0.04	Dig. < Conv.
Alfouzan et al., 2023 [29]	Milled (IvoBase)	7.7 × 10^3^ ± 5.8 × 10^3^	HPAR	14.3 × 10^3^ ± 13.1 × 10^3^	Dig. = Conv.
	3D-printed (NextDent)	5.0 × 10^3^ ± 5.8 × 10^3^			
Khattar et al., 2023 [32]	3D-printed (NextDent)	123.3 × 10^4^ ± 48.9 × 10^4^	HPAR	86 × 10^4^ ± 45.31 × 10^4^	Dig. = Conv.
Koujan et al., 2022 [27]	Milled (AvaDent)	0.90 ± 0.14	HPAR	1.04 ± 0.15	Dig. = Conv.
	3D-printed (Lucitone)	1.79 ± 0.13			Dig. > Conv.
Larijani et al., 2022 [33]	Milled (Glazed)	0.12 ± 0.02	HPAR	0.15 ± 0.02	Dig. = Conv.
	Milled (Glazed)	0.13 ± 0.01	HPAR	0.13 ± 0.02	
	Milled (Glazed)	0.12 ± 0.02	HPAR	0.13 ± 0.02	
	Milled (Glazed)	0.14 ± 0.02	HPAR	0.12 ± 0.01	
	Milled (High polished)	0.08 ± 0.01	HPAR	0.15 ± 0.02	Dig. < Conv.
	Milled (High polished)	0.09 ± 0.02	HPAR	0.13 ± 0.02	
	Milled (High polished)	0.12 ± 0.02	HPAR	0.13 ± 0.02	
	Milled (High polished)	0.07 ± 0.02	HPAR	0.12 ± 0.01	
Osman et al., 2023 [39]	Milled (Opera)	0.05 ± 0.004	HPAR	0.10 ± 0.02	Dig. < Conv.
	3D-printed (NextDent)	0.22 ± 0.02			Dig. > Conv.
Teixeira et al., 2023 [40]	3D-printed (Cosmos)	4.96 ± 0.43	HPAR	4.47 ± 0.60	Dig. > Conv.

### Qualitative results

The included 14 studies revealed variable results. Four studies [22, 23, 35, 36] showed significantly lower candida colonization on the digital dentures. Three studies [29, 31, 32] reported comparable results. Three studies [28, 30, 39] showed lower candida colonization on the milled digital dentures, but significantly higher candida colonization on the 3D-printed group as compared to the conventional dentures. Two studies [27, 40] reported higher candida colonization in the 3D-printed group than in the conventional group, but one of them reported no significant differences between the milled digital and the conventional groups. One study [33] reported lower candida colonization in the polished milled digital group compared to the conventional group, but no difference was noted between the glazed milled and the conventional group. One study [34] found no significant differences between the milled group and the conventional group, but it revealed significantly lower candida colonization in the 3D-printed groups compared to the conventional (Tables 1 and 2).

### Meta-analysis results

**Fig. 2:**
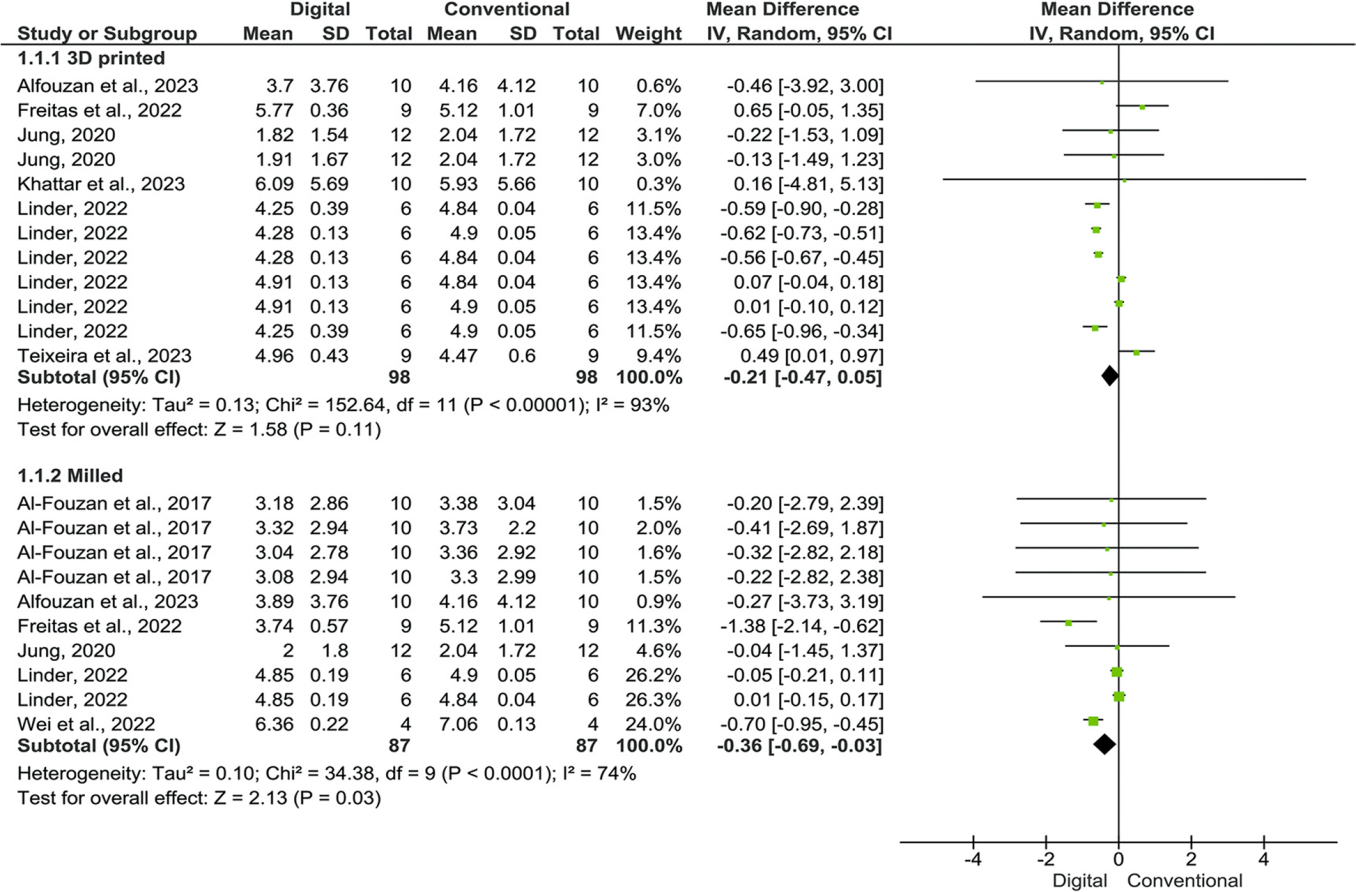
Forest plot of the pooled data for the included studies that used log CFU/ml as measurement unit

Figure 2 shows the meta-analysis model of studies that used CFU/ml as a measurement method of the outcome. There were six studies with 12 independent comparison groups that used the 3D-printed acrylic resins and six studies with 10 independent comparison groups that used the milled acrylic resins. The pooled data regarding comparing the 3D-printed versus the heat-polymerized acrylic resins revealed lower but non-significant candida colonization on the former compared to the latter (MD = − 0.21; 95%CI = − 0.47, 0.05; *P* = 0.11). However, the pooled data regarding comparing the milled vs. heat-polymerized acrylic resins revealed significant lower candida colonization on the former compared to the latter (MD = − 0.36; 95%CI = − 0.69, − 0.03; *P* = 0.03). Owing to the high heterogeneity amongst the studies (I^2^ = 89%; *P* < 0.00001), the random-effect model was used.

**Fig. 3:**
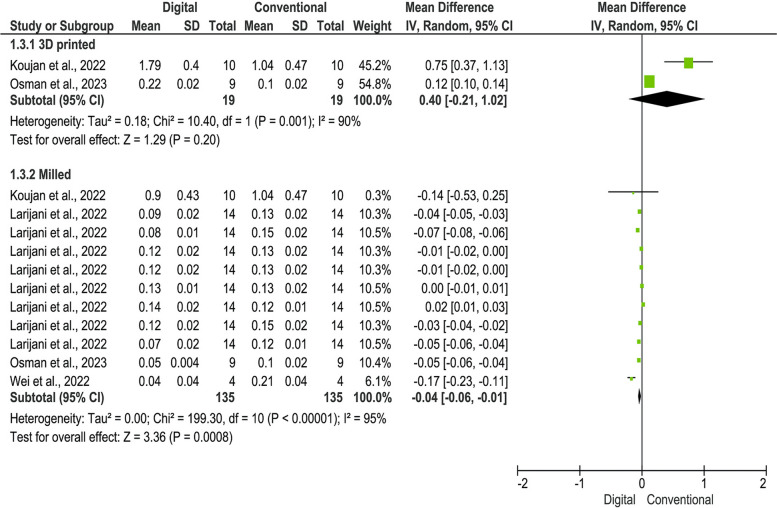
Forest plot of the pooled data for the included studies that used OD value as measurement unit

Figure 3 shows the meta-analysis model of studies that used OD value as a measurement method of the outcome. There were only two studies with two independent comparison groups that used the 3D-printed acrylic resin. The results of these studies revealed insignificant but higher candida colonization on the 3D-printed acrylic resin compared to the heat-polymerized acrylic resin (MD = 0.40; 95%CI = − 0.21, 1.02; *P* = 0.11). There were four studies with 11 independent comparison groups that used the milled acrylic resin. The pooled data revealed a significantly lower candida colonization in the milled resin group compared to the heat-polymerized acrylic resin group (MD = − 0.04; 95%CI = − 0.06, − 0.01; *P* = 0.0008). Owing to the high heterogeneity amongst the studies (I^2^ = 97%; *P* < 0.00001), the random-effect model was used.

### Publication Bias

**Fig. 4:**
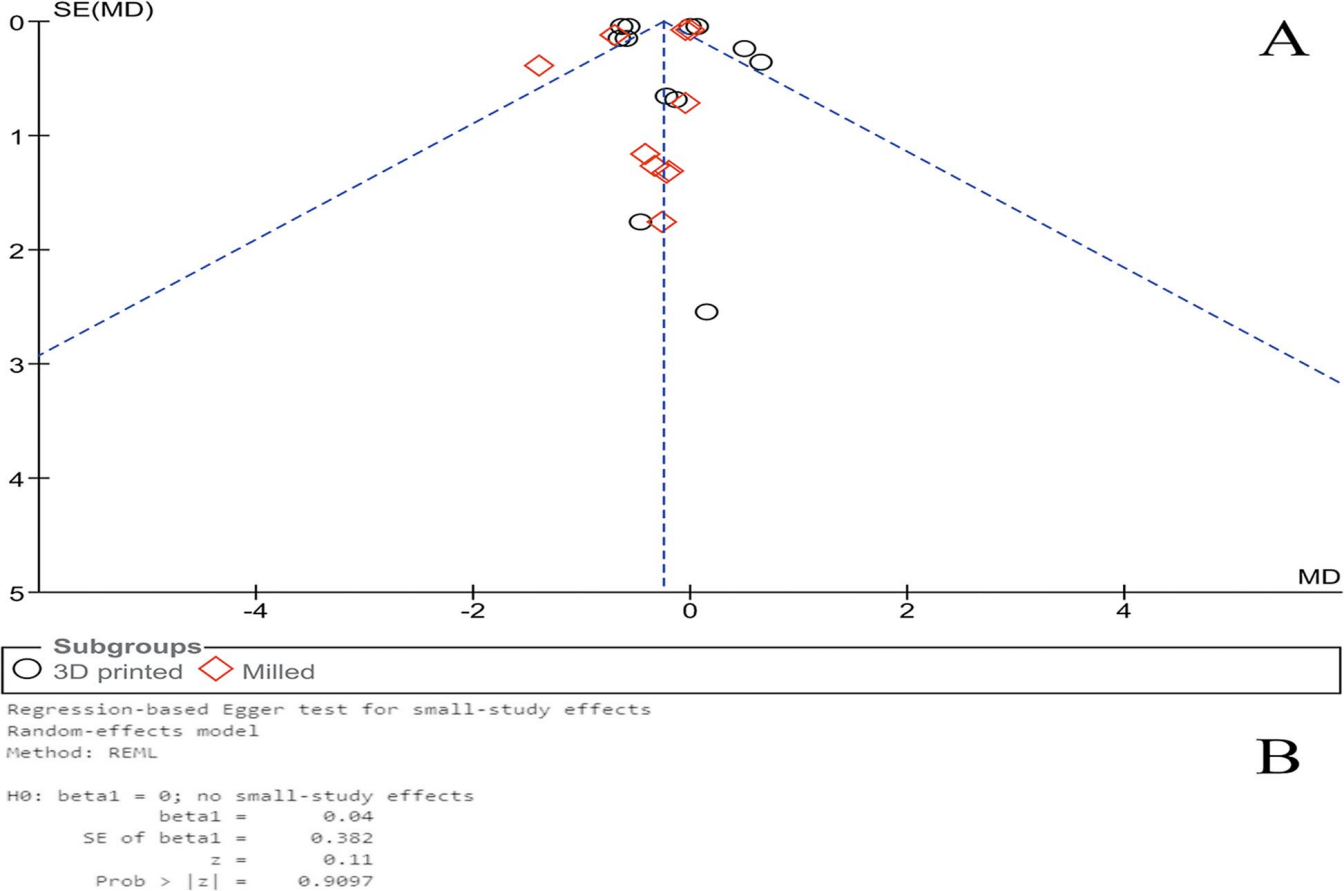
Publication bias (**A**) and Egger’s test (**B**) for the included studies that used log CFU/ml as measurement unit

**Fig. 5:**
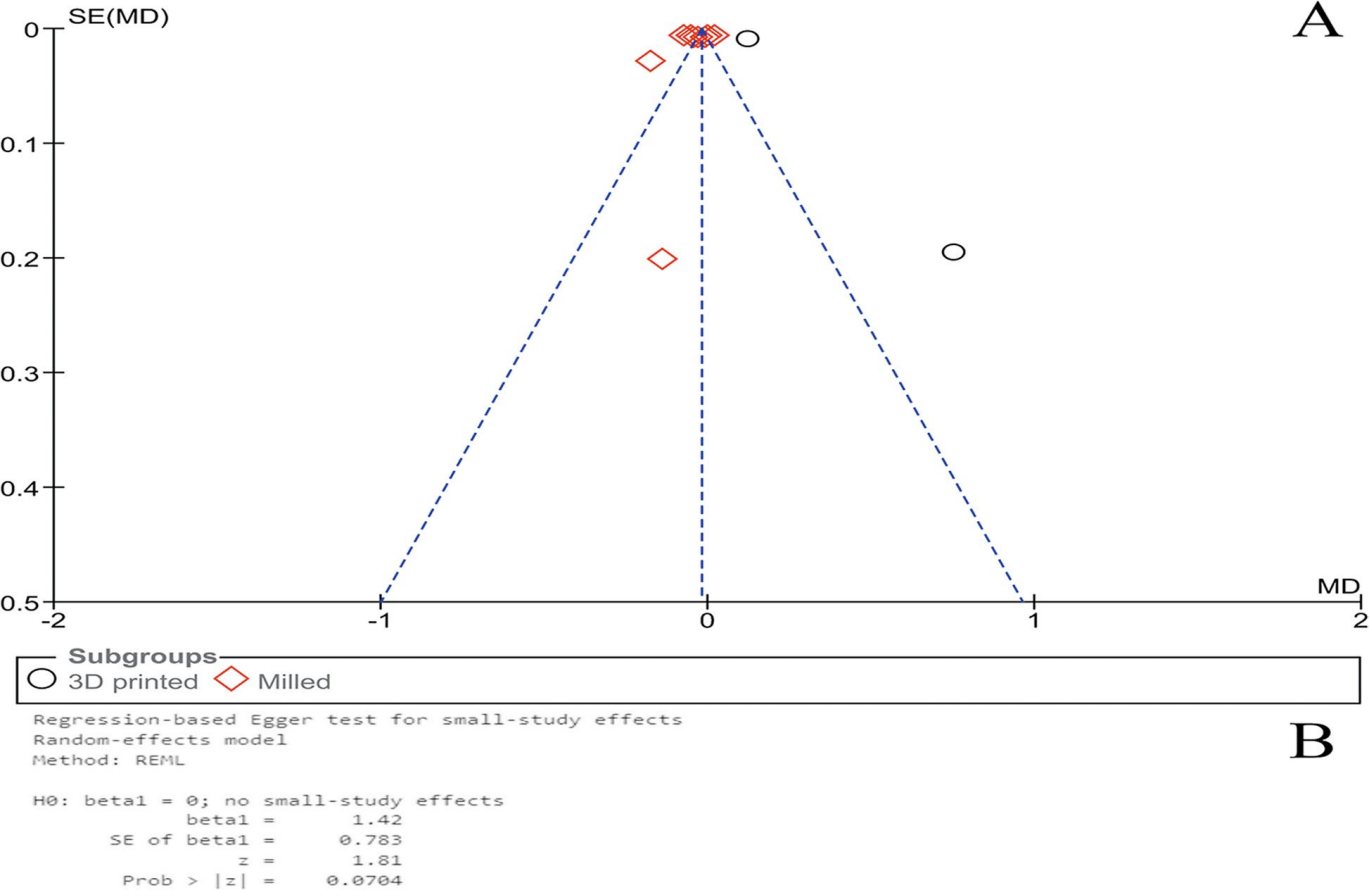
Publication bias (**A**) and Egger’s test (**B**) for the included studies that used OD value as measurement unit

As shown in Fig. 4, the qualitative (Fig. 4A) and quantitative (Fig. 4B) analyses revealed no publication bias among the studies that used CFU/ml (*P* = 0.910, Egger’s test). Similarly, regarding the studies that used OD value, no publication bias was found (Fig. 5A and B) (*P* = 0.070, Egger’s test).

### Quality of the included studies

**Fig. 6:**
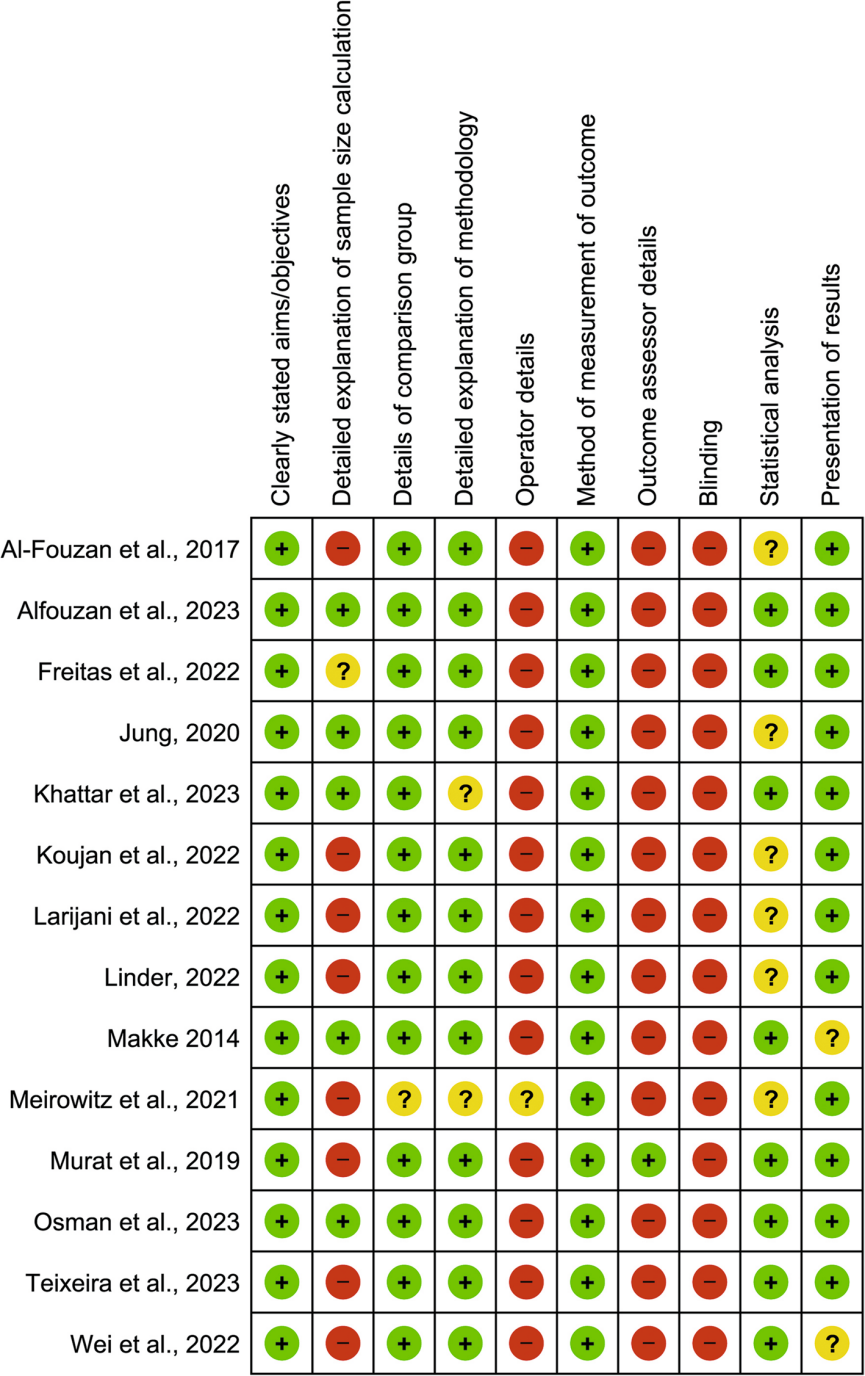
Risk of bias summary of the included studies

**Fig. 7:**
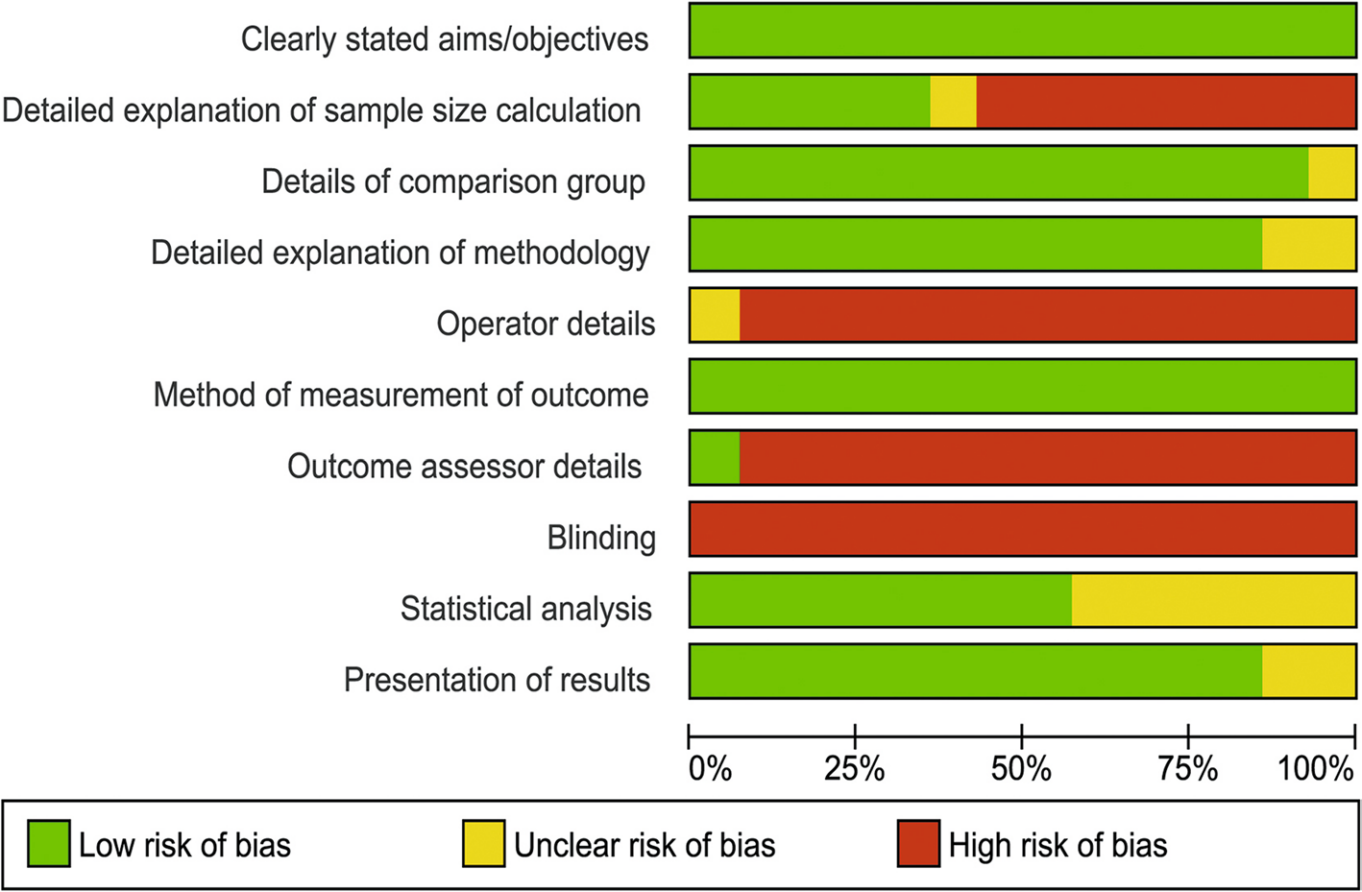
Risk of bias graph of the included studies

The quality results of the included studies are presented in Figs. 6 and 7. All the studies included in this systematic review and meta-analysis were found to be of moderate quality, ranging from 50 to 70%. The majority of the methodological shortcomings were related to sample size calculation, operator details, outcome assessor details, and blinding of the operator(s), outcome assessor(s), and statistician. Details of the statistical part were also missing from some studies.

## Discussion

While conventional materials and methods have long been used in denture fabrication, recent advances in dental materials and technologies have led to the development of new denture materials and digital fabrication methods [41–43]. However, these new materials and methods must demonstrate improved biocompatibility, better mechanical properties, and a less favourable environment for microbial adhesion in order to gain acceptance among dental professionals. In this review, we aimed to systematically summarize the available in-vitro evidence on the potential adhesion of *C. albicans* to digitally-fabricated acrylic resin materials in comparison to conventional ones. The key findings of our meta-analyses indicate that the adhesion of *C. albicans*, as measured by CFU or OD values, is lower on the milled digitally-fabricated resin materials compared to the conventionally-fabricated resin materials. This suggests that these digitally-fabricated materials either provide a less favourable environment for *C. albicans* colonization, or have anti-fungal properties. Regardless of the mechanism, this fact must be emphasized, disseminated among dental professionals, and incorporated into clinical practice.

As we have included studies that compared the same material in both conventional and digital methods, the proposed explanation that the digitally-fabricated resin materials have anti-fungal properties is negated. Yildirim et al. [44] concluded that the adhesion of *C. albicans* to of the resin surface may be influenced by the physicochemical properties more than the surface roughness. Instead, the mechanical properties of the materials are suspected to be the reason for the difference in *C. albicans* adhesion. These mechanical properties vary between the conventionally- and the digitally-fabricated dentures, with the latter providing a less favourable environment for *C. albicans* colonization. Recent systematic reviews have confirmed that many mechanical properties are different between the two methods of denture fabrication, with surface roughness being a key factor that may influence the adhesion of *C. albicans*. These reviews have shown that the conventionally-fabricated resins have higher surface roughness values than the digitally-fabricated resins [45–47]. Moreover, the surface roughness values of the conventionally-fabricated resins were found to be above the threshold value of 0.2 μm [48]. In a recent meta-analysis, three factors were identified as responsible for *C. albicans* adhesion to denture materials, including surface roughness, wettability (measured as contact angle), and surface free energy [49]. Al-Fouzan et al. [22] stated that “the rougher the surface, the greater the Candida colonization will be*.*” However, there is a significant debate regarding the effects of these factors [50–53]. Other factors including continued denture wearing, poor cleaning of dentures, non-brushing of tongues, as well as sleeping with dentures are also contributing factors to *C. albicans* adhesion to dentures [14, 54].

Given that the surface porosity and roughness of the acrylic resin are considered responsible factors for *C. albicans* adhesion, Gauch et al. [55] argued that denture surfaces could be considered an infection source. In fact, many studies have reported a strong positive correlation between *C. albicans* adhesion and the surface roughness of denture base polymers [23, 56], although one recent study contradicted such results [40]. Based on many previous scanning electron microscope studies, the conventionally-fabricated resin showed a more porous surface and multiple surface irregularities than CAD/CAM-fabricated resin [23, 36]. The presence of such irregularities, porosity, and/or imperfections on a given surface of a dental device enhances microbial accumulation, even when it is clean [57]. As mentioned earlier, a threshold of ≤0.2 μm is recommended in order to prevent the formation of biofilm on any dental hard and prosthetic surfaces [48].

The findings of the present review are important from a clinical point of view. The milled dentures often have smoother and more polished surfaces than conventionally-fabricated dentures. This, in turn, reduces the potential for Candida adherence. Furthermore, milled dentures are less porous and more resistant to moisture absorption compared to the conventionally-fabricated dentures, potentially reducing the favourable conditions for Candida growth. Moreover, better retention and stability are expected due to the precision and improved fit of the milled dentures. Collectively, this helps to reduce the likelihood of micro-movements that may cause friction and irritation, which in turn may create opportunities for Candida to colonize and adhere.

The current systematic review has both strengths and weaknesses. One of the strengths was focusing on in-vitro studies. Including all studies since inception date was another point of strength. Updating the search and conducting manual search in the references of the potential studies was a strong point ensuring covering all the potential studies. Also, the review included studies that assessed one type of material using different fabrication technologies and provided information on the effects of fabrication rather than solely on material types or ingredients. The tool used to assess the quality of the included studies is also robust, representing a strength aspect of the study. The quality of all included studies was moderate. Thereby, the overall evidence of the current study might be affected (downgraded). Accordingly, following a thorough, standardized, precise, and detailed methodology is highly recommended in future studies. However, one of the limitations of this review was that it only included studies published in English, which may have missed valuable information in other languages. Although the qualitative and quantitative tests, Funnel plot and Egger’s test, respectively, revealed no publication bias, this cannot be considered conclusive, especially with a few studies included. Therefore, the potential publication bias might be considered to exist, which represents one of the limitations of the current study. The heterogeneity among the included studies was relatively high. This heterogeneity might be due to the different sample sizes, different dimensions and shapes of the specimens, or the different exposure times to the candida culture among the included studies. Additionally, we tried our best to extract the numerical data from some of the included studies using the relevant software or formula; however, there were three studies that lacked the adopted quantitative data, and thus were excluded from the meta-analysis, which in turn could limit the conclusive evidence of this review. Yet, owing to some methodological limitations of the included studies, more robust in-vitro studies, along with well-designed clinical studies are highly recommended to discern the available evidence.

## Conclusion

In conclusion, the limited available evidence suggests that the milled digitally-fabricated acrylic resins provide less adhesion environment to *C. albicans* compared to the conventionally-fabricated materials. Moreover, in cases where a digitally-fabricated denture is the preferred choice, the recommendation leans toward the milled denture over the 3D-printed one.

### Supplementary Information

**Supplementary Material 1.**

**Supplementary Material 2.**

**Supplementary Material 3.**

## Data Availability

All data generated or analyzed during this study are already included.

## Abbreviations

PRISMA: Preferred Reporting Items for Systematic Review and Meta-analyses
*C. albicans*: *Candida Albicans*
DS: Denture-related Stomatitis
CFU: Colony Forming Unit
OD: Optical Density
CAD/CAM: Computer-Aided Design/Computer-Aided Manufacturing
MD: Mean Difference
CI: Confidence Interval
QUIN: Quality Assessment Tool For In Vitro Studies
ID: Identification
MS: Microsoft
SPSS: The Statistical Package for the Social Sciences

## References

[1] Ishida Y, Kuwajima Y, Kobayashi T, Yonezawa Y, Asack D, Nagai M, Kondo H, Ishikawa-Nagai S, Da Silva J, Lee SJ (2022). Current implementation of digital dentistry for removable prosthodontics in US dental schools. Int J Dent..

[2] Grant GT, Campbell SD, Masri RM, Andersen MR (2021). Glossary of digital dental terms, 2nd edition: American College of Prosthodontists and ACP Education Foundation. J Prosthodont.

[3] Kattadiyil MT, Goodacre CJ, Baba NZ (2013). CAD/CAM complete dentures: a review of two commercial fabrication systems. J Calif Dent Assoc.

[4] Villias A, Karkazis H, Yannikakis S, Theocharopoulos A, Sykaras N, Polyzois G (2021). Current status of digital complete dentures technology. Prosthesis.

[5] Schweiger J, Stumbaum J, Edelhoff D, Güth JF (2018). Systematics and concepts for the digital production of complete dentures: risks and opportunities. Int J Comput Dent.

[6] Srinivasan M, Chien EC, Kalberer N, Alambiaga Caravaca AM, Castelleno AL, Kamnoedboon P, Sauro S, Özcan M, Müller F, Wismeijer D (2022). Analysis of the residual monomer content in milled and 3D-printed removable CAD-CAM complete dentures: an in vitro study. J Dent.

[7] Alhallak K, Hagi-Pavli E, Nankali A. A review on clinical use of CAD/CAM and 3D printed dentures. Br Dent J. 2023. Epub ahead of print. 10.1038/s41415-022-5401-5. 10.1038/s41415-022-5401-5 36624309

[8] Mubaraki MQ, Moaleem MMA, Alzahrani AH, Shariff M, Alqahtani SM, Porwal A, Al-Sanabani FA, Bhandi S, Tribst JPM, Heboyan A, et al. Assessment of Conventionally and Digitally Fabricated Complete Dentures: A Comprehensive Review. Materials (Basel, Switzerland). 2022;15(11). 10.3390/ma15113868 PMC9182039 35683165

[9] Bidra AS, Taylor TD, Agar JR (2013). Computer-aided technology for fabricating complete dentures: systematic review of historical background, current status, and future perspectives. J Prosthet Dent.

[10] Kraemer Fernandez P, Unkovskiy A, Benkendorff V, Klink A, Spintzyk S. Surface characteristics of milled and 3D printed Denture Base materials following polishing and coating: an in-vitro study. Materials (Basel, Switzerland). 2020;13(15). 10.3390/ma13153305 PMC7435723 32722240

[11] Goodacre BJ, Goodacre CJ (2022). Additive manufacturing for complete denture fabrication: a narrative review. J Prosthodont.

[12] Gendreau L, Loewy ZG (2011). Epidemiology and etiology of denture stomatitis. J Prosthodont.

[13] Al-Maweri SA, Al-Jamaei AA, Al-Sufyani GA, Tarakji B, Shugaa-Addin B (2015). Oral mucosal lesions in elderly dental patients in Sana'a, Yemen. J Int Soc Prev Community Dent.

[14] Mousa MA, Lynch E, Kielbassa AM (2020). Denture-related stomatitis in new complete denture wearers and its association with Candida species colonization: a prospective case-series. Quintessence Int (Berlin, Germany : 1985).

[15] Ramage G, Tomsett K, Wickes BL, López-Ribot JL, Redding SW (2004). Denture stomatitis: a role for Candida biofilms. Oral Surg Oral Med Oral Pathol Oral Radiol Endod.

[16] Yarborough A, Cooper L, Duqum I, Mendonça G, McGraw K, Stoner L (2016). Evidence regarding the treatment of denture stomatitis. J Prosthodont.

[17] Arutyunov S, Kirakosyan L, Dubova L, Kharakh Y, Malginov N, Akhmedov G, Tsarev V. Microbial adhesion to dental polymers for conventional, computer-aided subtractive and additive manufacturing: a comparative in vitro study. J Funct Biomater. 2022;13(2). 10.3390/jfb13020042 PMC9036260 35466224

[18] Di Fiore A, Meneghello R, Brun P, Rosso S, Gattazzo A, Stellini E, Yilmaz B (2022). Comparison of the flexural and surface properties of milled, 3D-printed, and heat polymerized PMMA resins for denture bases: an in vitro study. J Prosthodont Res.

[19] Schubert A, Bürgers R, Baum F, Kurbad O, Wassmann T. Influence of the manufacturing method on the adhesion of Candida albicans and Streptococcus mutans to Oral splint resins. Polymers. 2021;13(10). 10.3390/polym13101534 PMC8150722 34064561

[20] Tsui C, Kong EF, Jabra-Rizk MA (2016). Pathogenesis of Candida albicans biofilm. Pathog Dis..

[21] Le Bars P, Kouadio AA, Bandiaky ON, Le Guéhennec L, de La Cochetière MF. Host's immunity and Candida species associated with denture stomatitis: a narrative review. Microorganisms. 2022;10(7). 10.3390/microorganisms10071437 PMC9323190 35889156

[22] Al-Fouzan AF, Al-Mejrad LA, Albarrag AM (2017). Adherence of Candida to complete denture surfaces in vitro: a comparison of conventional and CAD/CAM complete dentures. J Adv Prosthodont..

[23] Murat S, Alp G, Alatalı C, Uzun M (2019). In vitro evaluation of adhesion of Candida albicans on CAD/CAM PMMA-based polymers. J Prosthodont : Off J Am Coll Prosthodont..

[24] Li P, Fernandez PK, Spintzyk S, Schmidt F, Beuer F, Unkovskiy A (2022). Effect of additive manufacturing method and build angle on surface characteristics and Candida albicans adhesion to 3D printed denture base polymers. J Dent.

[25] Shim JS, Kim JE, Jeong SH, Choi YJ, Ryu JJ (2020). Printing accuracy, mechanical properties, surface characteristics, and microbial adhesion of 3D-printed resins with various printing orientations. J Prosthet Dent.

[26] Yacob N, Ahmad NA, Safii SH, Yunus N, Razak FA. Is microbial adhesion affected by the build orientation of a 3-dimensionally printed denture base resin? J Prosthet Dent. 2023; (In Press). 10.1016/j.prosdent.2023.04.017 37210224

[27] Koujan A, Aggarwal H, Chen PH, Li Z, Givan DA, Zhang P, Fu CC. Evaluation of Candida albicans adherence to CAD-CAM milled, 3D-printed, and heat-cured PMMA resin and efficacy of different disinfection techniques: an in vitro study. J Prosthodont : Off J Am Coll Prosthodont. 2022;32. 10.1111/jopr.13583 35941701

[28] Meirowitz A, Rahmanov A, Shlomo E, Zelikman H, Dolev E, Sterer N. Effect of Denture Base fabrication technique on Candida albicans adhesion in vitro. Materials (Basel, Switzerland). 2021;14(1). 10.3390/ma14010221 PMC7795816 33466383

[29] Alfouzan AF, Tuwaym M, Aldaghri EN, Alojaymi T, Alotiabi HM, Taweel SMA, Al-Otaibi HN, Ali R, Alshehri H, Labban N. Efficacy of denture cleansers on microbial adherence and surface topography of conventional and CAD/CAM-processed Denture Base resins. Polymers. 2023;15(2). 10.3390/polym15020460 PMC9866049 36679340

[30] Freitas R, Duarte S, Feitosa S, Dutra V, Lin WS, Panariello BHD, Carreiro A (2023). Physical, mechanical, and anti-biofilm formation properties of CAD-CAM milled or 3D printed Denture Base resins: in vitro analysis. J Prosthodont : Off J Am Coll Prosthodont..

[31] Jung GK (2020). In vitro analysis of attachment of *Candida Albicans* to Denture Base acrylic resins fabricated by three different methods.

[32] Khattar A, Alghafli JA, Muheef MA, Alsalem AM, Al-Dubays MA, AlHussain HM, AlShoalah HM, Khan SQ, AlEraky DM, Gad MM. Antibiofilm activity of 3D-printed nanocomposite resin: impact of ZrO(2) nanoparticles. Nanomaterials (Basel, Switzerland). 2023;13(3). 10.3390/nano13030591 PMC9921268 36770550

[33] Larijani M, Zareshahrabadi Z, Alhavaz A, Hajipour R, Ranjbaran A, Giti R, Soltankarimi V, Zomorodian K (2022). Evaluation of Candida albicans biofilm formation on conventional and computer-aided-design/computer-aided manufacturing (CAD/CAM) denture base materials. Curr Med Mycol..

[34] Linder WE (2022). Evaluation of adherence of *Candida albicans* to differently manufactured acrylic resin Denture Base materials.

[35] Aa M (2014). Comparison of *Candida Albicans* adhesion to various Denture Base materials.

[36] Wei X, Gao L, Wu K, Pan Y, Jiang L, Lin H, Wang Y, Cheng H (2022). In vitro study of surface properties and microbial adhesion of various dental polymers fabricated by different manufacturing techniques after thermocycling. Clin Oral Investig.

[37] Moher D, Liberati A, Tetzlaff J, Altman DG (2009). Preferred reporting items for systematic reviews and meta-analyses: the PRISMA statement. PLoS Med.

[38] Sheth VH, Shah NP, Jain R, Bhanushali N, Bhatnagar V. Development and validation of a risk-of-bias tool for assessing in vitro studies conducted in dentistry: The QUIN. J Prosthet Dent. 2022:S0022-3913(22)00345-6. 10.1016/j.prosdent.2022.05.019 35752496

[39] Osman RB, Khoder G, Fayed B, Kedia RA, Elkareimi Y, Alharbi N. Influence of fabrication technique on adhesion and biofilm formation of Candida albicans to conventional, milled, and 3D-printed Denture Base resin materials: a comparative in vitro study. Polymers. 2023;15(8). 10.3390/polym15081836 PMC10146129 37111983

[40] Teixeira ABV, Valente M, Sessa JPN, Gubitoso B, Schiavon MA, Dos Reis AC (2023). Adhesion of biofilm, surface characteristics, and mechanical properties of antimicrobial denture base resin. J Adv Prosthodont..

[41] Schweiger J, Edelhoff D, Güth JF. 3D printing in digital prosthetic dentistry: an overview of recent developments in additive manufacturing. J Clin Med. 2021;10(9). 10.3390/jcm10092010 PMC8125828 34067212

[42] Anadioti E, Musharbash L, Blatz MB, Papavasiliou G, Kamposiora P (2020). 3D printed complete removable dental prostheses: a narrative review. BMC Oral Health.

[43] Mohd Farid DA, Zahari NAH, Said Z, Ghazali MIM, Hao-Ern L, Mohamad Zol S, Aldhuwayhi S, Alauddin MS. Modification of polymer based dentures on biological properties: current update, status, and findings. Int J Mol Sci. 2022;23(18). 10.3390/ijms231810426 PMC9499318 36142344

[44] Yildirim MS, Hasanreisoglu U, Hasirci N, Sultan N (2005). Adherence of Candida albicans to glow-discharge modified acrylic denture base polymers. J Oral Rehabil.

[45] de Oliveira Limírio JPJ, Gomes JML, Alves Rezende MCR, Lemos CAA, Rosa C, Pellizzer EP (2022). Mechanical properties of polymethyl methacrylate as a denture base: conventional versus CAD-CAM resin - a systematic review and meta-analysis of in vitro studies. J Prosthet Dent.

[46] Srinivasan M, Kamnoedboon P, McKenna G, Angst L, Schimmel M, Özcan M, Müller F (2021). CAD-CAM removable complete dentures: a systematic review and meta-analysis of trueness of fit, biocompatibility, mechanical properties, surface characteristics, color stability, time-cost analysis, clinical and patient-reported outcomes. J Dent.

[47] Jain S, Sayed ME, Shetty M, Alqahtani SM, Al Wadei MHD, Gupta SG, Othman AAA, Alshehri AH, Alqarni H, Mobarki AH, et al. Physical and mechanical properties of 3D-printed provisional crowns and fixed dental prosthesis resins compared to CAD/CAM milled and conventional provisional resins: a systematic review and Meta-analysis. Polymers. 2022;14(13). 10.3390/polym14132691 PMC9269394 35808735

[48] Bollen CM, Lambrechts P, Quirynen M (1997). Comparison of surface roughness of oral hard materials to the threshold surface roughness for bacterial plaque retention: a review of the literature. Dent Mater : Off Publ Acad Dent Mater..

[49] Gad MM, Abualsaud R, Khan SQ (2022). Hydrophobicity of Denture Base resins: a systematic review and Meta-analysis. J Int Soc Prev Community Dent..

[50] Pereira-Cenci T, Cury AA, Cenci MS, Rodrigues-Garcia RC (2007). In vitro Candida colonization on acrylic resins and denture liners: influence of surface free energy, roughness, saliva, and adhering bacteria. Int J Prosthodont.

[51] Nevzatoğlu EU, Ozcan M, Kulak-Ozkan Y, Kadir T (2007). Adherence of Candida albicans to denture base acrylics and silicone-based resilient liner materials with different surface finishes. Clin Oral Investig.

[52] Hahnel S, Ettl T, Gosau M, Rosentritt M, Handel G, Bürgers R (2010). Influence of saliva substitute films on the initial adhesion of Candida albicans to dental substrata prior to and after artificial ageing. Arch Oral Biol.

[53] de Foggi CC, Machado AL, Zamperini CA, Fernandes D, Wady AF, Vergani CE (2016). Effect of surface roughness on the hydrophobicity of a denture-base acrylic resin and Candida albicans colonization. J Investig Clin Dent.

[54] Mousa MA, Alam MK, Ganji KK, Khader Y, Lynch E, Kielbassa AM (2022). Prospective case series on possible effects of local factors on the development of halitosis in new complete denture wearers. Quintessence Int (Berlin, Germany : 1985)..

[55] Gauch LMR, Pedrosa SS, Silveira-Gomes F, Esteves RA, Marques-da-Silva SH (2018). Isolation of Candida spp. from denture-related stomatitis in Pará, Brazil. Braz J Microbiol : [publ Braz Soc Microbiol]..

[56] da Silva WJ, Leal CM, Viu FC, Gonçalves LM, Barbosa CM, Del Bel Cury AA (2015). Influence of surface free energy of denture base and liner materials on Candida albicans biofilms. J Investig Clin Dent.

[57] Verran J, Maryan CJ (1997). Retention of Candida albicans on acrylic resin and silicone of different surface topography. J Prosthet Dent.

