# Supplementary figures and images for: Adhesion of Candida Albicans to digital versus conventional acrylic resins: a systematic review and meta-analysis
Source: BMC Oral Health. 2024 Mar 4;24:303. doi: 10.1186/s12903-024-04083-2 (PMC10910815; doi:10.1186/s12903-024-04083-2)

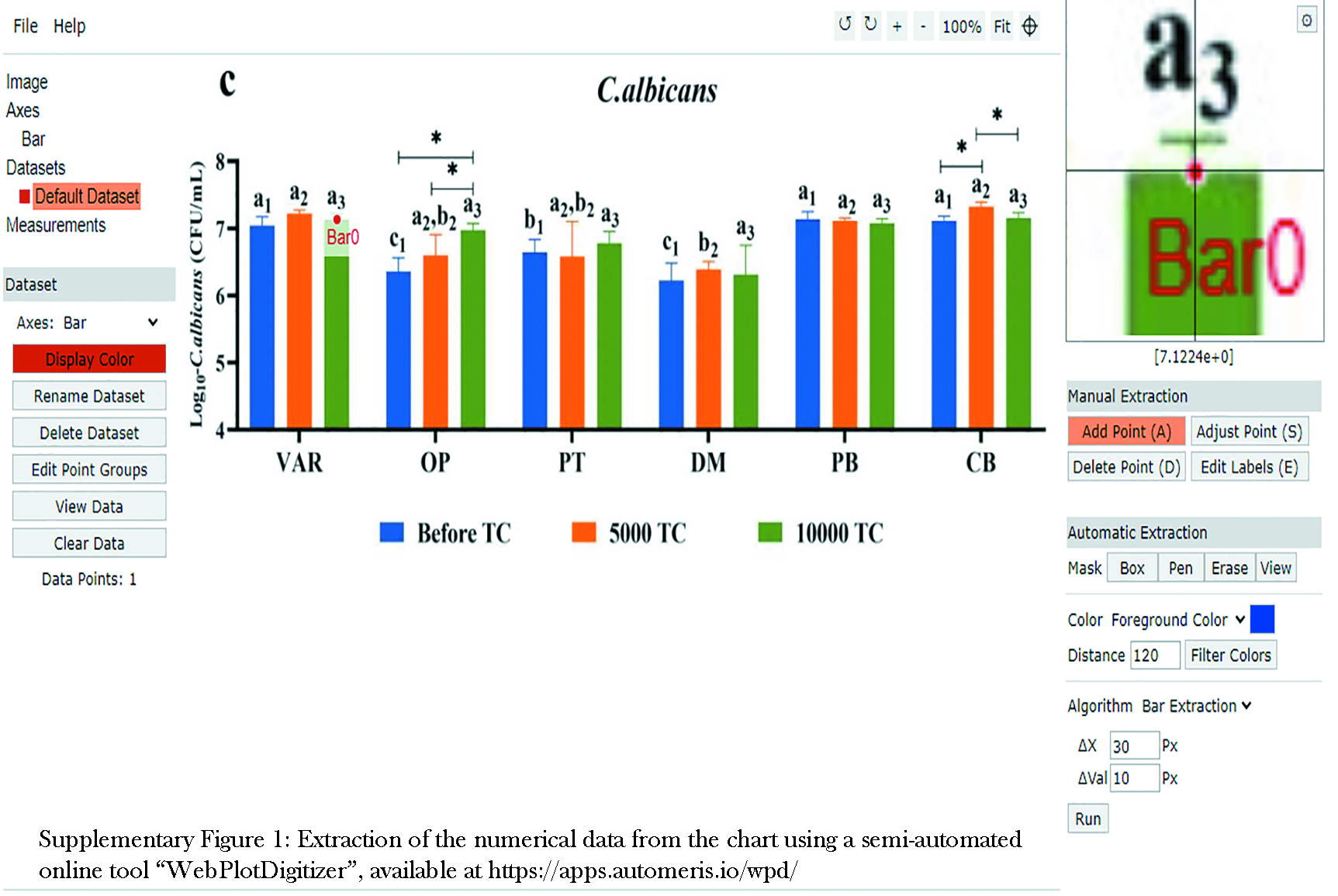

Supplement: Supplementary file 1 — Supplementary Material 1. [file 12903_2024_4083_MOESM1_ESM.jpg]
