# Supplementary material for: Adhesion of Candida Albicans to digital versus conventional acrylic resins: a systematic review and meta-analysis
Source: BMC Oral Health. 2024 Mar 4;24:303. doi: 10.1186/s12903-024-04083-2 (PMC10910815; doi:10.1186/s12903-024-04083-2)
Supplement: Supplementary file 2 — Supplementary Material 2. [file 12903_2024_4083_MOESM2_ESM.docx]

**Supplementary Table 1. Databases: Applied search strategy, and numbers of retrieved studies**

| Databases | Search strategy used | Hits |
| --- | --- | --- |
| PubMed (advanced search) was searched on January 9, 2023 and updated on May 29, 2023.  <https://pubmed.ncbi.nlm.nih.gov/advanced> | (("CAD-CAM denture"[Title/Abstract]) OR ("CAD/CAM denture"[Title/Abstract]) OR ("digital denture"[Title/Abstract]) OR ("3d printed denture"[Title/Abstract]) OR ("printed denture"[Title/Abstract]) OR ("printed resin"[Title/Abstract]) OR ("milled denture"[Title/Abstract]) OR ("milled resin"[Title/Abstract]) OR ("conventional heat-polymerized acrylic resin"[Title/Abstract]) OR ("conventional resins"[Title/Abstract]) OR ("conventional denture"[Title/Abstract]) OR ("heat-polymerized acrylic resin"[Title/Abstract]) OR ("heat-polymerized acrylic denture"[Title/Abstract])) AND (("antibacterial"[Title/Abstract]) OR ("antimicrobial"[Title/Abstract]) OR ("antifungal"[Title/Abstract]) OR ("adhesion"[Title/Abstract]) OR ("candida"[Title/Abstract]) OR ("colonization"[Title/Abstract])) | 80 |
| ISI web of science Core Collection (advanced search) was searched on January 9, 2023 and updated on May 29, 2023.  <https://www.webofscience.com/wos/woscc/advanced-search> | TS=("CAD-CAM denture" OR "CAD/CAM denture" OR "digital denture" OR "3d printed denture" OR "printed denture" OR "printed resin" OR "milled denture" OR "milled resin" OR "conventional heat-polymerized acrylic resin" OR "conventional resins" OR "conventional denture" OR "heat-polymerized acrylic resin" OR "heat-polymerized acrylic denture") AND TS=("antibacterial" OR "antimicrobial" OR "antifungal" OR "adhesion" OR "candida" OR "colonization") | 99 |
| Scopus was searched on January 9, 2023 and updated on May 29, 2023.  <https://www.scopus.com/search/form.uri?display=advanced> | TITLE-ABS-KEY("CAD-CAM denture" OR "CAD/CAM denture" OR "digital denture" OR "3d printed denture" OR "printed denture" OR "printed resin" OR "milled denture" OR "milled resin" OR "conventional heat-polymerized acrylic resin" OR "conventional resins" OR "conventional denture" OR "heat-polymerized acrylic resin" OR "heat-polymerized acrylic denture") AND TITLE-ABS-KEY("antibacterial" OR "antimicrobial" OR "antifungal" OR "adhesion" OR "candida" OR "colonization") | 165 |
| Ovid (advanced search, all sources) was searched on January 9, 2023 and updated on May 29, 2023.  <https://ovidsp.dc1.ovid.com/ovid-b/ovidweb.cgi> | ("CAD-CAM denture" OR "CAD/CAM denture" OR "digital denture" OR "3d printed denture" OR "printed denture" OR "printed resin" OR "milled denture" OR "milled resin" OR "conventional heat-polymerized acrylic resin" OR "conventional resins" OR "conventional denture" OR "heat-polymerized acrylic resin" OR "heat-polymerized acrylic denture") AND ("antibacterial" OR "antimicrobial" OR "antifungal" OR "adhesion" OR "candida" OR "colonization") | 53 |
| Google Scholar was searched on January 9, 2023 and updated on May 29, 2023.  <https://scholar.google.com/schhp?hl=en>  The first 10 pages (n= 100 records) were retrieved. | ("CAD-CAM denture" OR "CAD/CAM denture" OR "digital denture" OR "3d printed denture" OR "printed denture" OR "printed resin" OR "milled denture" OR "milled resin" OR "conventional heat-polymerized acrylic resin" OR "conventional resins" OR "conventional denture" OR "heat-polymerized acrylic resin" OR "heat-polymerized acrylic denture") AND ("antibacterial" OR "antimicrobial" OR "antifungal" OR "adhesion" OR "candida" OR "colonization") | 100 |
| Total |  | 497 |

**Supplementary Table 2: List of excluded studies and the reason of exclusion**

| # | **Reference** | **Reason for exclusion** |
| --- | --- | --- |
| **1** | Aati S, Aneja S, Kassar M, Leung R, Nguyen A, Tran S, Shrestha B, Fawzy A. Silver-loaded mesoporous silica nanoparticles enhanced the mechanical and antimicrobial properties of 3D printed denture base resin. Journal of the mechanical behavior of biomedical materials. 2022 Oct 1;134:105421. | No conv. acrylic resin |
| **2** | Aati S, Chauhan A, Shrestha B, Rajan SM, Aati H, Fawzy A. Development of 3D printed dental resin nanocomposite with graphene nanoplatelets enhanced mechanical properties and induced drug-free antimicrobial activity. Dental Materials. 2022 Dec 1;38(12):1921-33. | No conv. acrylic resin |
| **3** | Aati S, Shrestha B, Fawzy A. Cytotoxicity and antimicrobial efficiency of ZrO2 nanoparticles reinforced 3D printed resins. Dental Materials. 2022 Aug 1;38(8):1432-42. | No conv. acrylic resin |
| **4** | Abualsaud R, Aleraky DM, Akhtar S, Khan SQ, Gad MM. Antifungal activity of denture base resin containing nanozirconia: In vitro assessment of candida albicans biofilm. The Scientific World Journal. 2021 Jan 1;2021. | No digital acrylic resin |
| **5** | Alzayyat ST, Almutiri GA, Aljandan JK, Algarzai RM, Khan SQ, Akhtar S, Matin A, Gad MM. Antifungal Efficacy and Physical Properties of Poly(methylmethacrylate) Denture Base Material Reinforced with SiO2 Nanoparticles. J Prosthodont. 2021 Jul;30(6):500-508. | No digital acrylic resin |
| **6** | Idriss H, Elashnikov R, Rimpelová S, Vokatá B, Haušild P, Kolská Z, Lyukatov O, Švorčík V. Printable Resin Modified by Grafted Silver Nanoparticles for Preparation of Antifouling Microstructures with Antibacterial Effect. Polymers (Basel). 2021 Nov 6;13(21):3838. | No conv. acrylic resin |
| **7** | Jeon S, Jo YH, Yoon HI, Han JS. Effect of phytochemical-filled microcapsules with antifungal activity on material properties and dimensional accuracy of denture base resin for three-dimensional printing. BMC Oral Health. 2022 May 13;22(1):178. | No conv. acrylic resin |
| **8** | Jeon S, Jo YH, Yoon HI, Han JS. Antifungal effect, surface roughness, and cytotoxicity of three-dimensionally printed denture base with phytoncide-filled microcapsules: An in-vitro study. J Dent. 2022 May;120:104098. | No conv. acrylic resin |
| **9** | Jo YH, Lee WJ, Lee JH, Yoon HI. Antifungal activity, mechanical properties, and accuracy of three-dimensionally printed denture base with microencapsulated phytochemicals on varying post-polymerization time. BMC Oral Health. 2022 Dec 15;22(1):611. | No conv. acrylic resin |
| **10** | Lee HE, Alauddin MS, Mohd Ghazali MI, Said Z, Mohamad Zol S. Effect of Different Vat Polymerization Techniques on Mechanical and Biological Properties of 3D-Printed Denture Base. Polymers (Basel). 2023 Mar 15;15(6):1463. | No conv. acrylic resin |
| **11** | Li P, Fernandez PK, Spintzyk S, Schmidt F, Beuer F, Unkovskiy A. Effect of additive manufacturing method and build angle on surface characteristics and Candida albicans adhesion to 3D printed denture base polymers. J Dent. 2022 Jan;116:103889. | No conv. acrylic resin |
| **12** | Li P, Fernandez PK, Spintzyk S, Schmidt F, Yassine J, Beuer F, Unkovskiy A. Effects of layer thickness and build angle on the microbial adhesion of denture base polymers manufactured by digital light processing. J Prosthodont Res. 2023 Epub ahead of print. | No conv. acrylic resin |
| **13** | Shim JS, Kim JE, Jeong SH, Choi YJ, Ryu JJ. Printing accuracy, mechanical properties, surface characteristics, and microbial adhesion of 3D-printed resins with various printing orientations. J Prosthet Dent. 2020 Oct;124(4):468-475. | No conv. acrylic resin |
| **14** | Yacob N, Ahmad NA, Safii SH, Yunus N, Razak FA. Is microbial adhesion affected by the build orientation of a 3-dimensionally printed denture base resin? J Prosthet Dent. 2023 (In Press). | No conv. acrylic resin |
